# P02.01. A dietary approach for treating dyslipidemia and hyperglycemia

**DOI:** 10.1186/1472-6882-12-S1-P57

**Published:** 2012-06-12

**Authors:** J Feuerstein, L Bautista, W Bjerke

**Affiliations:** 1 Stamford Hospital, Columbia University, Stamford, USA; 2 Optimus Healthcare, Stamford, USA; 3 Sacred Heart University, Fairfield, USA

## Purpose

Elevated LDL cholesterol and impaired fasting glucose are significant risk factors for cardiovascular disease; the most prevalent cause of mortality in the USA. Many dietary approaches have been examined to help combat these medical problems. Each type of diet typically places a particular emphasis on the relative proportions of the three macronutrients; fat, carbohydrate and protein.

## Methods

We report on a case series of 41 patients who were placed on an 1100-calorie diet reduced in starch and emphasizing lean proteins, mono and polyunsaturated fats and fiber with a unique composition of macronutrients for four months in an effort to improve cholesterol and fasting glucose indices.

## Results

28 of the 41 (68%) patients complied with the protocol over a four-month period. In the compliant group, statistically significant reduction (p<0.05) in the following mean variables were seen: Weight (2.3kg), Total Cholesterol 22% (53 mg/dL), LDL 23% (43 mg/dL), HDL (4 mg/dL), TAG (21 mg/dL) and fasting serum glucose (12 mg/dL), after 4 months on the dietary regimen. In the non-compliant/comparison group, statistically significant increases (p<0.05) in the following variables were seen at the end of 6 months: Total Cholesterol (24 mg/dL), LDL (14 mg/dL) and TAG (29 mg/dl).

## Conclusion

This magnitude of reduction in Total and LDL Cholesterol is significantly greater than that seen in the recent large dietary intervention trials and is comparable to that seen in the 'eco-Atkins' trial, which was far more restrictive in nature and shorter in duration.

